# Supplementary material for: Dynamic Changes in the Nutrient Digestibility, Rumen Fermentation, Serum Parameters of Perinatal Ewes and Their Relationship with Rumen Microbiota
Source: Animals (Basel). 2024 Aug 14;14(16):2344. doi: 10.3390/ani14162344 (PMC11350810; doi:10.3390/ani14162344)
Supplement: Supplementary file 1 [file animals-14-02344-s001.zip › animals-3153169-supplementary.pdf]

Table S1. Sequence analysis and statistics of rumen microbiota in perinatal ewes

Item	Q21	Q14	Q7	H3	H7	H14
raw tags	549,685	440,575	625,649	523,052	654,850	450,986
clean tags	541,273	433,267	612,634	511,626	638,914	440,954
clean tags/ ewe	90,212	72,211	102,106	85,271	106,486	73,492
OTUs	2,250	2,287	2,278	2,082	2,037	2,205

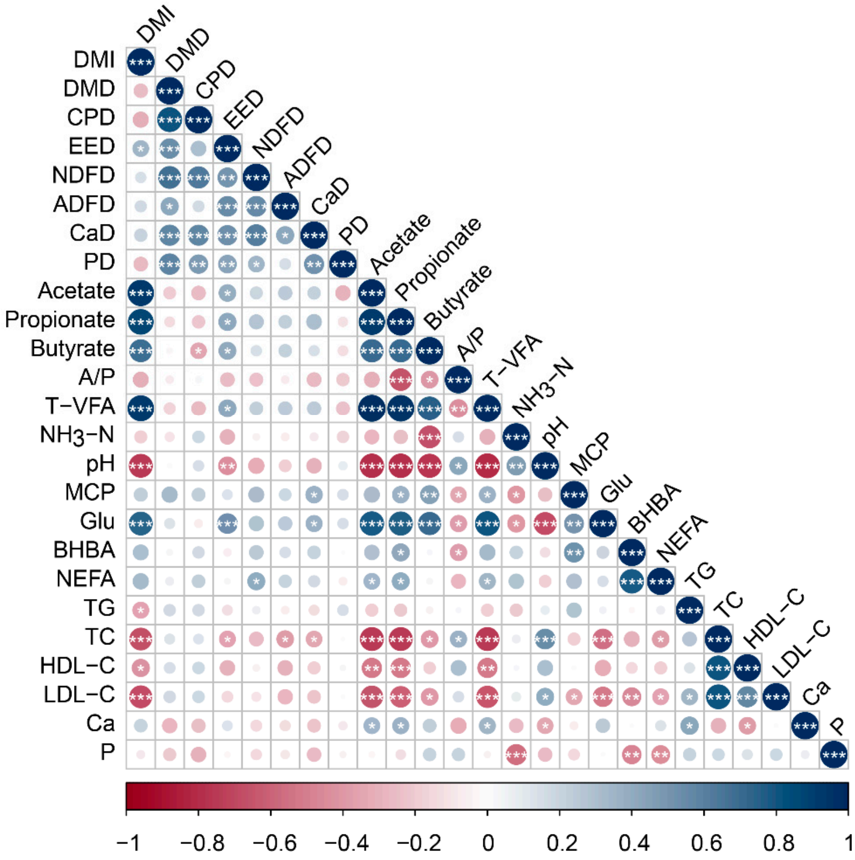

Figure S1. Correlation between dry matter intake, dietary digestibility, rumen fermentation parameters and serum biochemical indices in perinatal ewes.

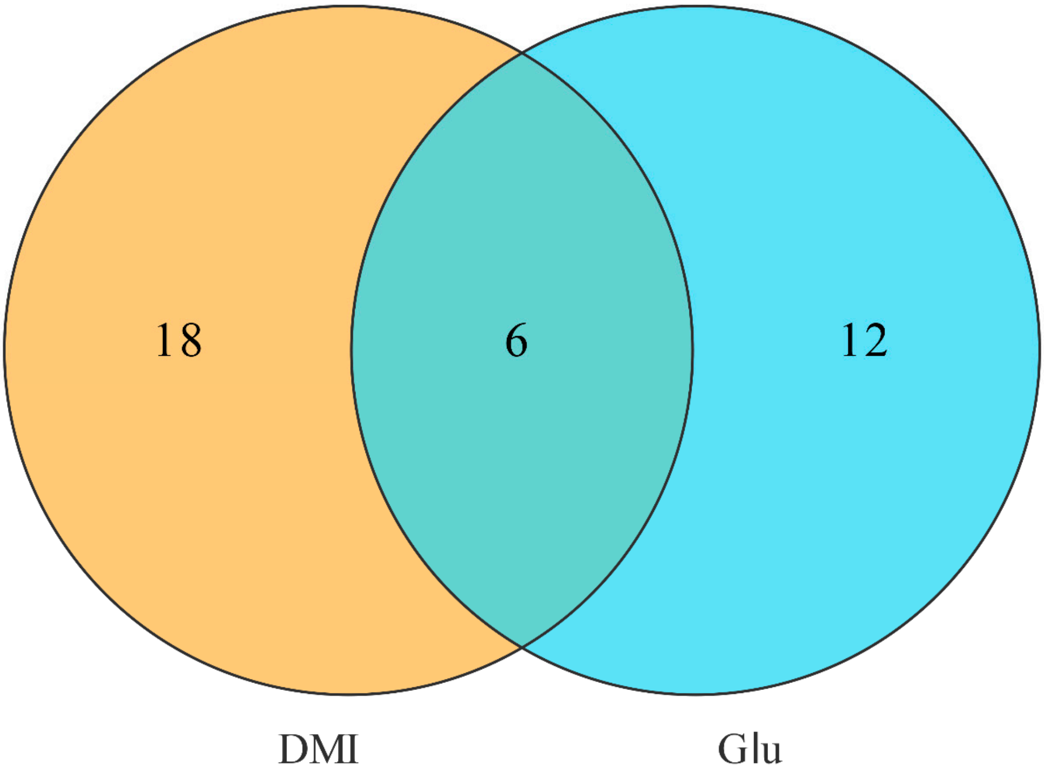

Figure S2. Venn diagram analysis of the DMI and Glu dominant microorganisms predicted by random forest.
